# Identification and Fine-Mapping of a Major Maize Leaf Width QTL in a Re-sequenced Large Recombinant Inbred Lines Population

**DOI:** 10.3389/fpls.2018.00101

**Published:** 2018-02-07

**Authors:** Baobao Wang, Yanbin Zhu, Jinjie Zhu, Zhipeng Liu, Han Liu, Xiaomei Dong, Jinjie Guo, Wei Li, Jing Chen, Chi Gao, Xinmei Zheng, Lizhu E, Jinsheng Lai, Haiming Zhao, Weibin Song

**Affiliations:** State Key Laboratory of Agrobiotechnology and National Maize Improvement Center, Department of Plant Genetics and Breeding, China Agricultural University, Beijing, China

**Keywords:** fine-mapping, maize, leaf width, QTL, *qLW4*, large RIL population, genotyping-by-sequencing (GBS)

## Abstract

Leaf width (LW) influences canopy architecture of population-cultured maize and can thus contribute to density breeding. In previous studies, almost all maize LW-related mutants have extreme effect on leaf development or accompanied unfavorable phenotypes. In addition, the identification of quantitative trait loci (QTLs) has been resolution-limited, with cloning and fine-mapping rarely performed. Here, we constructed a bin map for 670 recombinant inbred lines (RILs) using ∼1.2 billion 100-bp re-sequencing reads. QTL analysis of the LW trait directly narrowed the major effect QTL, *qLW4*, to a ∼270-kb interval. A fine-mapping population and near-isogenic lines (NILs) were quickly constructed using a key RIL harboring heterozygous genotypes across the *qLW4* region. A recombinant-derived progeny testing strategy was subsequently used to further fine-map *qLW4* to a 55-kb interval. Examination of NILs revealed that *qLW4* has a completely dominant effect on LW, with no additional effect on leaf length. Candidate gene analysis suggested that this locus may be a novel LW controlling allele in maize. Our findings demonstrate the advantage of large-population high-density bin mapping, and suggest a strategy for efficiently fine-mapping or even cloning of QTLs. These results should also be helpful for further dissection of the genetic mechanism of LW variation, and benefit maize density breeding.

## Introduction

Leaf is the most important organ for photosynthesis, which is the process of providing most carbohydrates for plant growth and development. Maize, one of the world’s most important crops as food, feed and the biomass material, has long, narrow, alternately arranged leaves with parallel veins. The width of leaves influences canopy architecture in population-cultured maize and thus affects photosynthetically active radiation and light signal transmission (Vogelmann and Gorton, 2014). On the one hand, excessively narrow leaves are benefit for light transparency, but greatly limit light capture (Pepper et al., 1977). On the other hand, the excessively wide one vastly reduce light transmitted to middle and lower leaves, thereby reduce the overall photosynthetically active radiation and cause shading avoidance, a syndrome results in lots of disadvantageous phenotype for maize development (Kebrom and Brutnell, 2007). The trade-off between LW and light capture or signal transmission is thus important for maize breeding, and an efficient tool is consequently needed to specifically manipulate LW without causing negative impacts. It was reported that yield improvement in modern maize breeding relies more strongly on adaptation to a higher plant density than on an increase in grain number per plant, with canopy structure having an important effect on density tolerance (Hammer et al., 2009). The identification of alleles controlling LW would thus not only be beneficial for understanding mechanisms of leaf development; it would also be helpful for the intentional modification of leaf architecture and therefore assist maize breeding, especially density breeding.

Previously, several genes and mutations had been found to be involved in maize leaf development and to affect maize LW. In particular, three genes have been reported to be directly involved in the control of LW variation. The duplicate genes *NS1* (*Narrow Sheath1*) and *NS2* (*Narrow Sheath2*) (Nardmann et al., 2004), which encode WUSCHEL-like homeobox genes, perform redundant functions in maize leaf development, with the extreme narrow sheath phenotype in maize caused by mutations at these two unlinked loci. The *CslD1* (*Cellulose Synthase-Like D1*) gene (Hunter et al., 2012), encoding a Cellulose Synthase-Like D protein, is involved in cell expansion and division. Mutation of this gene can cause a 35% reduction in LW and lead to other narrow-organ and warty phenotypes. Besides, several genes taking part in maize leaf development also affect LW variation. *LBL1* (*LeafBladeless1*) (Nogueira et al., 2007) and *RGD2* (*Ragged Seedling2*) (Douglas et al., 2010), the genes involved in biogenesis of *trans*-acting small interfering RNA, impact leaf polarity establishment and thus influence phenotypes associated with narrowed leaves. Regulation of *LGN1* (*Liguleless Narrow1*) and *SLN1* (*Sister of Liguleless Narrow1*) (Moon et al., 2013), which are protein kinase-encoding genes, influence the establishment of positional boundaries and result in liguleless and narrow leaf phenotypes. *DIL1* (*Dwarf* and *Irregular Leaf1*) (Jiang et al., 2012), an AP2 transcription factor-like gene, simultaneously influences plant height and LW and length. Mutation of *Asc1* (*asceapen1*) (Brooks et al., 2009), a maize D4-cyclin gene, also causes an obvious narrow phenotype in maize leaves. Furthermore, several mutations that are yet to be cloned exhibit an influence on maize LW; these include *wab1* (*Wavy auricles in blades1*) (Hay and Hake, 2004), *ffm1* (*filifolium1*) (Mellor and Langdale, 2009), *nl1* (*narrow leaf1*) and *nl2* (*narrow leaf2*) (Neuffer and Sheridan, 1977). Unfortunately, all of these mutations have extreme effect on leaf development or unfavorable pleiotropic phenotypic effects, which limit their application to maize breeding.

In addition to being controlled by a wide range of qualitative genes, maize LW is affected by minor-polygenes (Tian et al., 2011), thus revealing its quantitative nature. Studies over past decades have identified LW QTLs dispersed across the 10 maize chromosomes (Reymond et al., 2004; Nikolic et al., 2011; Tian et al., 2011; Ku et al., 2012; Zheng and Liu, 2013; Guo et al., 2015). However, the confidence intervals of these detected QTLs were relatively large, ranging from ∼2 to 17 cM (Reymond et al., 2004; Nikolic et al., 2011; Tian et al., 2011; Ku et al., 2012; Zheng and Liu, 2013; Guo et al., 2015). This poor precision has hindered physical mapping, and the intervals are too large to define candidate genes. Furthermore, LW QTL cloning or fine-mapping work has rarely been performed up to now.

As is well known, successful QTL mapping is limited by marker density and population size. Advances in sequencing technology have facilitated the use of high-throughput SNPs and Indel markers for this purpose. One successful example is bin mapping, a method that joint redundancy SNPs and Indels as bin markers for linkage mapping (Huang et al., 2009). In rice and sorghum, this method had already been conducted for QTL mapping. The accuracy was proved better than traditional PCR-based markers, and many cloned genes and QTLs could be re-mapped, confirming its practicability and resource-saving (Huang et al., 2009; Yu et al., 2011; Zou et al., 2012). However, its precision and creditability was still skeptical because of the little population size (Vales et al., 2005), which could even lead to misleading inferences. Previously, it has been reported that the large population size could improve the number, accuracy, and precision of QTL detection (Schön et al., 2004). Cloning of *ZmCCT* in maize nested association mapping population (NAM, harboring nearly 5000 RILs) (Hung et al., 2012), as well as cloning of *qSN8* and *qSPB1* in an enlarged rice RILs population (1709 lines) by PCR markers (Gao et al., 2013), and verification of *Scmv1* in two large maize RILs populations (2206 lines) (Tao et al., 2013a) further implied feasibility of fine-mapping or cloning major effect QTLs in only one large, high-resolution genotyped RIL population.

In this study, we conducted QTL mapping for LW using a large maize RILs population, which was genotyped by GBS method. A major effect QTL, *qLW4*, was directly mapped to 270 kb interval and further narrowed down to 55 kb by progenies derived from a key RIL harboring heterozygous genotypes across the *qLW4* region. Effect and candidate genes for *qLW4* were discussed. These findings may shed light on efficiently QTL fine mapping in maize, be beneficial for further cloning of *qLW4*, and provide molecular markers for maize breeding.

## Materials and Methods

### RILs Planting and Phenotypic Evaluations

The population used for QTL identification comprised 883 RILs and was derived by single-seed descent from a cross between X178 and HuangC. X178 and HuangC are the parents of Nongda108, a very important maize hybrid in China. LW phenotypes were collected for F_6:7_ RILs at two locations in China: Sanyuan (40°05′ N, 116°12′ E) in Beijing in 2010 and Xinxiang (35°20′ N, 113°43′ E) in Henan Province in 2012. A randomized complete block design with two replications was used for trials in Sanyuan, while trials in Xinxiang followed an augmented incomplete block design (Tian et al., 2011) with a single replication. One-row plots were used in all trials; the plot size was 3 m, with 13 plants per row and a 0.5-m row spacing, resulting in a final plant density of 80,000 plants ha^-1^. LWs were measured from the widest region of the first leaf above the uppermost ear of 10 (Sanyuan) or 5 (Xinxiang) plants per plot. After harvesting of ears in Xinxiang, cob color was recorded as white or red, which was represented in subsequent analyses as 1 and 2, respectively. LW data were modeled with a mixed linear model using the *lme4* package in R (Bates et al., 2014) as follows: LW = Line + Loc + Year + Line:Loc + Line:Year + error, where Line is the RIL, Loc is location, Year is year, Line:Loc is the interaction of genotype and location, and Line:Year is the genotype-year interaction. The heritability (*h*^2^) of LW was calculated in *R* according to the following formula: *h*^2^= var(Line)/[var(Line) + var(Line:Loc)/*e* + var(Line:Year)/ *r*+var(Residual)/*re*], where var represents variance, *e* is the number of locations, and *r* is the number of years.

### RIL DNA Isolation, Sequencing, SNP Identification, and Bin Map Construction

Samples for DNA extraction consisted of bulked shoot tissue collected from six or more seedlings per genotype. The CTAB protocol was used for DNA extraction.

X178 and HuangC were sequenced at coverage depths of 5× and 2.5×, respectively, as previously reported (Jiao et al., 2012). GBS library construction and sequencing were performed as described in Chen et al. (2014).

Single nucleotide polymorphisms identification was carried out according to a previously described pipeline (Chen et al., 2014). Briefly, read alignment was conducted in BWA using version 2 of the B73 maize reference genome, and SNP detection was performed with the Genome Analysis Toolkit. Only SNPs with uniquely identified map positions were retained.

To ensure map quality in the current analysis, we only retained RILs with sequence coverage depths larger than 0.03× and more than 20,000 genotyped SNPs. As a result, 24 RILs were excluded.

A bin map was constructed followed a modified sliding window method with a window size of 20 SNPs and a slide step of 2 SNPs (Song et al., 2016). RILs with more than 10% residual heterozygosity or more than 200 breakpoints (124 RILs) were excluded from the subsequent analysis to avoid potential genotyping errors. After this step, 670 RILs remained. Heterozygous genotypes were then set as missing and imputed using the “argmax” method as implemented in the R/qtl package. Bins with a distorted segregation ratio larger than 2/1 or a heterozygous ratio larger than 10% were deleted. Linkage map distances were calculated by the “Kosambi” method using the *est.map* function in R/qtl.

### QTL Analysis of Nongda108 RILs

Quantitative trait loci analysis of the Nongda108 RILs was performed using the R/qtl package as described in Song et al. (2016). Where QTL detection, LOD threshold calculation, QTL confidence interval definition, and QTL effect evaluation were, respectively, carried out using the composite interval mapping method, the 1,000 permutations (*p* < 0.05) approach, the 1.5 LOD-drop method, and a linear QTL model.

### Verification of *qLW4*

To verify *qLW4*, 74 RILs with recombination breakpoints in ∼1 Mb downstream and upstream flanking regions of the *qLW4* interval (according to the bin map) were selected and genotyped with 10 PCR-based markers. The five Indel (Indel-0.832, Indel-1.459, Indel-2.836, Indel-3.89, and Indel-4.1) and five Cleaved Amplified Polymorphic Sequences (Caps-2.815, Caps-3.215, Caps-3.406, Caps-3.442, and Caps-3.618) markers were designed using Primer5 and SNP2CAPS software, respectively. In 2013, the 74 RILs were additionally phenotyped for LW in Sanya (18°23′ N, 109°10′ E), Hainan Province, where they were grown using the same field design as in Xinxiang. A simple Student’s *t*-test was used to assess whether a marker was associated with LW on the basis of the -log_10_ transformed *p*-values obtained.

### NILs Identification and Map-Based Fine-Mapping of *qLW4*

Taking advantage of the large mapping population, we examined genotypes of all Nongda108 RILs and uncovered an F_6:7_ RIL, named R1504, with a heterozygous bin map genotype in the *qLW4* region. This RIL was further verified by the presence of segregated genotypes at the *qLW4* locus in its F_7_ progenies. Selfing of F_7_ progenies of R1504 in the winter of 2013 (Sanya; 18°23′ N, 109°10′ E) yielded two NILs, named W964 (wider leaves and with the X178 genotype in the *qLW4* region) and W968 (narrower leaves and with the HuangC genotype in the *qLW4* region). The two NILs were subsequently genotyped by the GBS method at a 0.06× coverage depth to check for genome-wide uniformity between them. SNP detection followed the same method mentioned above, except that only those SNPs covered by more than two reads were retained. In addition, 17 Indels (chr1-3.1, chr1-202.2, chr2-126.4, chr2-196.2, chr3-226.1, chr4-98.3, chr4-222.7, chr5-76.6, chr6-25.3, chr6-145.9, chr8-25.3, chr8-40.8, chr8-120.6, chr9-1.465, chr9-155.4, chr10-104.2, and chr10-142.1) distributed among all 10 chromosomes except for chromosome 7 were chosen for the same analysis. In the summer of 2014 (Shangzhuang; 40°08′ N, 116°11′ E), the width of all mature leaves of the two parents (X178 and HuangC) and the two NILs (W964 and W968) were measured, and a W964 × W968 cross was performed. In the winter of 2014 (Sanya; 18°23′ N, 109°10′ E), the width, length, and parallel venation number of the widest region of the ear leaf were measured for W964, W968, and W964/W968.

In the summer of 2014, all progenies of R1504 were planted in Shangzhuang (40°08′ N, 116°11′ E), which ultimately yielded 1,128 plants. Each plant was labeled, sampled, and subjected to DNA extraction as described above. The width of ear leaf (LW0), 1st upper leaf (LW1) and 2nd upper leaf (LW2) of each plant were measured. Breakpoints around the QTL interval were identified by genotyping these samples with PCR-based markers (Caps_3.255, Indel_3.256, Caps_3.399, Caps_3.406, Caps_3.413, Caps_3.442, Indel_3.451, Indel_3.453, Indel_3.459492, Indel_3.459603, Indel_3.459816, Indel_3.4664 75, Indel_3.467037, Indel_3.467651, Indel_3.467953, Indel_3.468 695, Indel_3.474215, Caps_3.584979, Caps_4-3.618 and Indel_ 3.74), which were designed with Primer5 and SNP2CAPS as described above. Recombinants with a heterozygous genotype on one side of the breakpoint (around the QTL interval) and homozygous on the other side were crossed with W968 (narrower leaves and with a homozygous HuangC genotype in the *qLW4* region). Progenies of these recombinants were planted in Sanya (18°23′ N, 109°10′ E) in the winter of 2014, followed by genotyping and phenotyping by the same method. In each recombinant, the *qLW4* region was divided into two segments: a heterozygous part and a homozygous part. The heterozygous segment was segregated into two types of genotype in its W968-crossed progenies: heterozygous and homozygous HuangC. In accordance with the recombinant-derived progeny testing strategy described by Yang et al. (2012), a simple *t*-test was then performed to assess the significance of the LW difference (*p* < 0.05) between these genotypes, to determine which side of the breakpoint *qLW4* was located. The resulting data were analyzed using Excel and R software.

### Quantitative Real-Time PCR (qPCR)

Samples used for qPCR were collected from plants at VE (when first leaf appeared) and V9 (when the ninth leaf collar emergence) stage. Seedlings of W964 and W968 were sown and grown in a growth chamber (28°C ± 2°C, 16-h light/8-h dark). The V9 plants were grown in winner of 2017 in Hainan. Samples from root and shoot of VE seedlings, immature tassel, developing leaf, mature leaf of V9 plants were collected. Three biological replicates were performed for each sample. Total RNA was extracted using the TRIzol reagent (Invitrogen, Cat# 15596018) after the samples were ground in liquid nitrogen. RNA quantity and quality were monitored by Agilent 2100 system (Agilent Technologies). cDNA was synthesized using M-MLV RT (Promega, Cat# M170) after the RQ1 RNase-Free Dnase treatment (Promega, Cat# M6101). qPCR was performed in ABI7500 instrument (Applied Biosystems) using SYBR Premix Ex Taq Mix (Takara, Cat # RR820A). *EF1α* was used as the internal reference gene.

## Results

### Sequencing, SNPs Detection, and Bin Map Construction and Validation

X178 and HuangC, the parents of Nongda108 RILs, were sequenced at 5× and 2.5× coverage depths, respectively. SNP identification via a strict pipeline yielded 1,743,421 SNPs between the two parents, of which ∼66 and 34% were located in intergenic and genic regions, respectively (Supplementary Figure S1). We then sequenced 818 of the 883 Nongda108 RILs using a GBS strategy. Available data was acquired for794 lines, resulting in ∼1.2 billion generated 100-bp reads with an average sequence depth of 0.08× (Supplementary Figure S2). After SNP calling, an average of 24,375 SNPs were obtained for each line, with a maximum of 36,424 and a minimum of 13,824. The average distance between adjacent SNPs was 102 kb.

**FIGURE 1:**
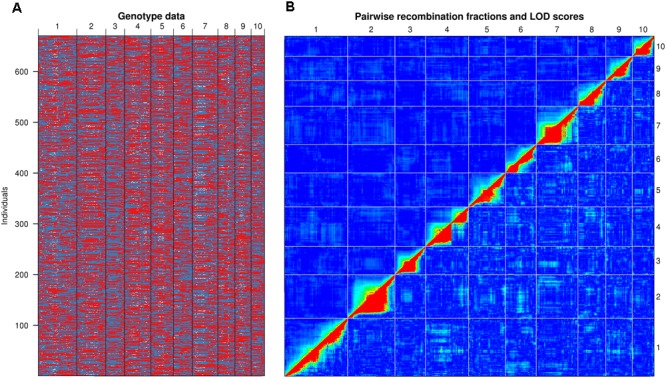
Bin map profile. **(A)** Graphical representation of genotypes of 670 Nongda108 RILs. Red, X178 genotype; blue, HuangC genotype; white, heterozygous. **(B)** Pairwise recombination fractions of bins. Upper left, recombination fractions between each pair of bins; lower right, logarithm (base 10) of odds (LOD) scores from linkage testing. Linked signals between bins are mainly located on a diagonal line, indicating no obvious problems with the bin map.

A bin map was generated by a modified sliding window method for a subgroup of 670 Nongda108 RILs (which excluded RILs with too much residual heterozygosity or breakpoints, see method). Totally, 23,843 bin markers were generated (**Figure 1A** and Supplementary Table S1). The physical distance between midpoints of adjacent bins ranged from 10 kb to 2.7 Mb, with an average of 91 kb (Supplementary Table S1). A genetic map with a length of 3230.45 cM was generated using R/qtl software. The largest linkage interval between adjacent bins was 15.119 cM, while the mean was 0.139 cM (Supplementary Table S1). Pair-wise recombination fractions were checked for all bins, and linkage signals were diagonally displayed (**Figure 1B**). This pattern was further verified by pair-wise linkage disequilibrium analysis (Supplementary Figure S3).

**FIGURE 2:**
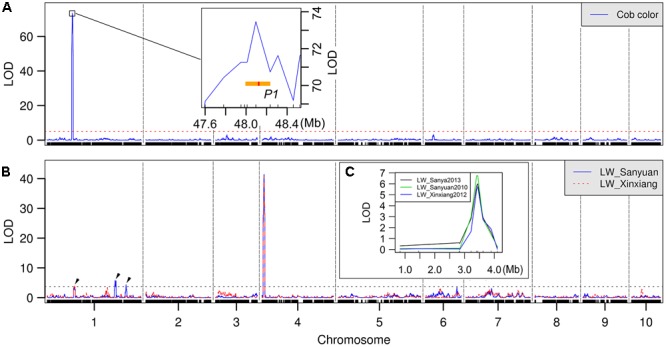
Quantitative trait loci (QTL) mapping profile. **(A)** QTL analysis of cob color in Nongda108 RILs. The *P1* gene and its tandem repeat region are enlarged in the display. **(B)** QTL analysis of LW in Nongda108 RILs at two locations. After filtering with an LOD cutoff of 3.62 (Sanyuan) and 3.69 (Xinxiang), four QTLs were finally detected. Only the lower cutoff of 3.62 for Sanyuan was shown. The three QTLs located on chromosome 1 were pointed out by arrows. **(C)** Verification of *qLW4* by PCR-based markers. The *y*-axis represents -log_10_ transformed *p*-values from *t*-testing.

Cob color, a well-studied trait in maize, was first used to validate bin map quality and the QTL mapping pipeline. As a putative qualitative trait, the cob color of 788 Nongda108 RILs in Xinxiang was recorded as either red or white in 2012. A red:white segregation ratio of 395:403 was obtained, very similar to the expected 1:1 ratio (chi-square test, *p* = 0.777). QTL mapping revealed that only one locus was responsible for this trait variation; it was located in the interval of 47.99–48.69 Mb on chromosome 1 (**Figure 2A**). The marker with the highest LOD score, bin1_802, spanning 48.06–48.16 Mb, was just co-localized with the cloned *Pericarp color1* (*P1*) gene (**Figure 2A**). The *P1* gene encodes an R2R3-MYB transcription factor, which is an important component involved in maize anthocyanin biosynthetic pathway, is known to control maize cob color (Lechelt et al., 1989). The exact mapping of the *P1* gene confirmed the accuracy of our data panel.

### QTL Analysis of the LW Trait in Nongda108 RILs

Leaf width phenotypic data of parents and Nongda108 RILs were collected during 2 years (Sanyuan in 2010 and Xinxiang in 2012). The female parent, X178, exhibited markedly wider leaves than male parent HuangC (Supplementary Figure S4). Transgressive segregation of the LW trait was observed in the RILs, while the mean of the population was found to be skewed toward the wider-leafed parent, X178 (Supplementary Figure S4). Variance component analysis using the *lme4* package in R returned a high heritability value, 0.893, for LW in the Nongda108 RILs (Supplementary Table S2).

The QTL analysis was conducted using the CIM function in R/qtl. With logarithm (base 10) of odds (LOD) cutoffs of 3.62 and 3.69 determined by a 1,000-iteration permutation test (*p* < 0.05) for LW in Sanyuan and Xinxiang, respectively, four QTLs were identified (**Figure 2B**). Three QTLs, designated as *qLW1a, qLW1b*, and *qLW1c*, were located on chromosome 1. The fourth QTL, *qLW4*, was identified on chromosome 4. Two positive alleles of these QTLs were from HuangC; the rest were from X178, with X178 mainly increasing LW and HuangC having the opposite effect (**Table 1**). The total effect of the X178 alleles was more than twofold larger than that of HuangC (additive effect, 0.52 + 1.41 vs. 0.40 + 0.44 cm), consistent with the contrasting LW phenotypes of the two parental lines (**Table 1**). *qLW1a* and *qLW4* were detected in both environments, reflecting the steady effect of these two loci. All QTLs on chromosome 1 were minor effect QTLs, having an explained phenotypic variation and additive effect of less than 2% and 0.6 cm, respectively. These chromosome-1 QTLs spanned relatively small intervals: 8.35 Mb (Chr1: 51.41–59.76 Mb) for *qLW1a*, 5.02 Mb (Chr1: 240.69–245.71 Mb) for *qLW1b*, and 2.87 Mb (Chr1: 263.05–265.92 Mb) for *qLW1c*. In contrast, *qLW4* was a putative major effect QTL: it had an LOD score greater than 40, explained ∼25% of the phenotypic variation, and could vary LW by more than 1.4 cm. To our excitement, the QTL interval of *qLW4* only spanned 0.27 Mb (Chr4: 3.28–3.55 Mb), suggesting the possibility of rapidly identifying the candidate gene for this QTL.

**Table 1:** Leaf width (LW) QTLs identified in Nongda108 RILs.

			Peak		QTL interval					
Location	QTL	Chr.^a^	Pos.(cM)	Pos.(Mb)^b^	Linkage (cM)	Physical (Mb)	LOD^c^	Var (%)^d^	Add.^e^	Positive allele
Sanyuan	*qLW1a*	1	152.96	55.02	147.94–157.98	51.41–59.76	3.89	1.38	0.40	HuangC
	*qLW1b*	1	382.01	241.13	381.26–388.15	240.69–245.71	5.76	1.68	–0.52	X178
	*qLW1c*	1	443.18	264.02	442.06–446.70	263.05–265.92	4.23	1.02	0.44	HuangC
	*qLW4*	4	11.89	3.41	10.83–12.71	3.28–3.52	41.43	25.47	–1.41	X178
Xinxiang	*qLW1a*	1	152.96	55.02	147.94–157.9	51.41–59.60	3.87	1.25	0.35	HuangC
	*qLW4*	4	11.89	3.41	10.83–13.01	3.28–3.55	40.76	24.81	–1.40	X178

*^*a*^Chromosome; ^*b*^positions in linkage map (unit: cM) or physical map (unit: Mb); ^*c*^logarithm (base 10) of odds for corresponding QTL peak; ^*d*^percentage of explained phenotypic variation by corresponding QTL; ^*e*^aAdditive effect of corresponding QTL.*

### Verification of *qLW4* by PCR Markers

Although GBS can yield robust genotype data suitable for QTL mapping, this method suffers from the drawbacks of high numbers of missing markers and limited genome coverage. The latter disadvantage will further influence the accuracy of QTL mapping. To compensate for this drawback, we therefore verified the major LW QTL, *qLW4*, using PCR-based markers. We checked the bin map data, selected 74 RILs harboring crossover points around the *qLW4* interval, and genotyped the RILs again using 10 PCR-based markers (Indel-0.832, Indel-1.459, Indel-2.836, Indel-3.89, Indel-4.1, Caps-2.815, Caps-3.215, Caps-3.406, Caps-3.442, and Caps-3.618). A simple *t*-test was performed to assess the relationship between LW and these markers. Sharp peaks with the highest LODs across three environments were found to be associated with Caps-3.406 (**Figure 2C** and Supplementary Table S3). This marker was located at 3.406 Mb on chromosome 4 (B73 reference genome V2) and just overlapped with the bin-mapped QTL peak, thus supporting the reliability of *qLW4*.

### Identification of Near-Isogenic Lines (NILs) and Fine-Mapping of *qLW4*

A large RIL population increases the possibility of acquiring RILs harboring heterozygous genotypes in the target region with the rest genome mostly homozygous. Taking this advantage into consideration, we screened all 794 sequenced F_6:7_ Nongda108 RILs. Excitingly, a RIL, named R1504, that was heterozygous in the *qLW4* region with relative little heterozygosity elsewhere, was eventually identified (Supplementary Figure S5). Two NILs, designated as W964 and W968, were generated by the selfing of R1504 in the winter of 2013 (Supplementary Figure S6). W964 was homozygous for the X178 genotype in the *qLW4* region and had much wider leaves than did W968, which was homozygous for the HuangC genotype in the *qLW4* region. W964 and W968 were further genotyped with 17 PCR markers and by the GBS method. The 17 PCR markers, which were scattered across all 10 chromosomes except for chromosome 7, were all similar between W964 and W968 (Supplementary Figure S6). GBS genotyping yielded 256 SNPs (polymorphic between HuangC and X178) that were covered by at least two sequencing reads, of which only 20 differed between the two NILs (Supplementary Figure S6). Thus, the total similarity between the two NILs was 92.7%.

All the F_7_ seed of R1504 was subsequently planted in Shangzhuang in the summer of 2014, which resulted in 1,128 plants in the field. Widths of the ear leaf, 1st and 2nd leaf upper ear were measured in the mature plants. Notably, the widths of these three leaves all exhibited bimodal distributions in the R1504 progenies, with the lower-width peaks clustered around the width of W968 and the higher ones centered around W964 (Supplementary Figure S7). Ratios of the number of individuals surrounding W964 vs. W968 were in accord with an approximate segregation ratio of 3:1 (chi-test, *p* = 0.129, 0.084, and 0.079 for the three respective leaves; Supplementary Figure S7), indicating the presence of a single dominant Mendelian factor contributing to LW variation in this population.

**FIGURE 3:**
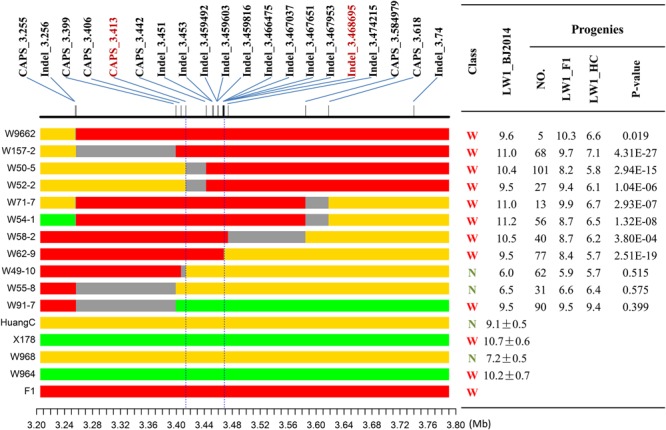
Fine-mapping of *qLW4*. Eleven recombinants are shown. Red, heterozygous; yellow, HuangC; green, X178; gray, unknown genotype. Each recombinant was divided into two types of segments in the *qLW4* region: a heterozygous part and a homozygous part. The widths of their 1st upper leaves in Shangzhuang in the summer of 2014 are shown. Each recombinant was further tested by crossing with W968, followed by analysis of the resulting progenies. The heterozygous segment of each recombinant was segregated into two types of genotypes in its W968-crossed progenies: heterozygous and homozygous HuangC. A simple *t*-test was performed to assess LW differences between these genotypes. If a significant difference (*p* < 0.05) was found, *qLW4* was located in the heterozygous region; otherwise, it was in the homozygous region. Finally, *qLW4* was mapped to a 55-kb region flanked by the markers Caps_3.413 and Caps_3.442.

Genotyping of progenies of R1504 with 20 PCR markers (see section “Materials and Methods”; Supplementary Table S4), uncovered several recombinants in the *qLW4* region in the summer of 2014. Eleven recombinants that were heterozygous for markers on one side of the *qLW4* region and homozygous on the other side were retained and subsequently crossed with the narrow-leafed NIL W968 (homozygous HuangC genotype in the *qLW4* region) to generate 11 F_7:8_ sub-families (**Figure 3**). In the winter of 2014, approximately 530 progenies of these 11 F_7:8_ sub-families were planted, phenotyped, and genotyped. In general, each W968-crossed F_7_ recombinant segregated into two types of genotypes with respect to the heterozygous portion of the *qLW4* locus in the F_7:8_ sub-families: homozygous HuangC and heterozygous genotypes. LW differences between these two genotypes in each sub-family were analyzed by *t*-testing. If a significant LW difference was observed between the two segregated genotypes in a F_7:8_ sub-family, the QTL was presumed to be situated in the non-homozygous region of its F_7_ progenitor; otherwise, it was presumed to reside in the non-heterozygous region. Three key recombinants representing two types of recombinations (**Figure 3**) were identified among the 11 recombinants. W50-5 and W52-2, both harboring a breakpoint between Caps_3.413 and Caps_3.442, exhibited a significant LW difference between the two segregated genotypes in their F_7:8_ sub-families; *qLW4* was thus localized to their shared non-homozygous regions, downstream of Caps_3.413. Using the same method, another key recombinant, W62-9, defined the location of this QTL to a region upstream of marker Indel_3.468695. With these three recombinants, *qLW4* was finally narrowed to a 55-kb region on chromosome 4 (3.4136–3.4687 Mb, B73 reference genome V2). The remaining eight recombinants supported this inference (**Figure 3**).

### Genetic Modeling and Effects of *qLW4* on LW

**FIGURE 4:**
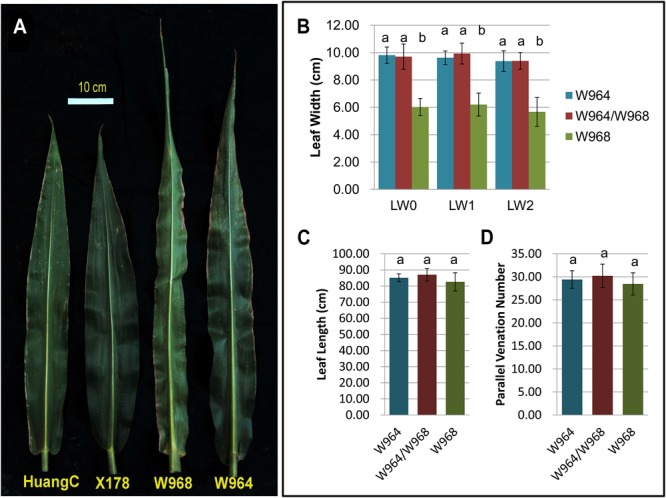
Effect of *qLW4*. **(A)** 1st upper leaves of HuangC, X178, W964, and W968. **(B)** Statistical analysis of widths of ear leaves (LW0), 1st upper leaves (LW1), and 2nd upper leaves (LW2) of W968, W964, and W964/W968 (*n* = 15). Significant differences were observed between LWs of W968 vs. W964 and W968 vs. W964/W968 but not W964 vs. W964/W968, indicating that *qLW4* has a completely dominant effect. **(C)** Statistical analysis of lengths of 1st upper leaves of W968, W964, and W964/W968 (*n* = 15). **(D)** Statistical analysis of parallel vein numbers of ear leaves of W968, W964, and W964/W968 (*n* = 15).

By comparing LWs of the two NILs and their F_1_ offspring, we found that the LWs of W964 and W964/W968 were both significantly wider than that of W968, whereas no significant difference was observed in LW between W964 and W964/W968. This result indicated that the effects of X178 and heterozygous alleles of *qLW4* were both significantly different from that of homozygous HuangC, but no significant effect difference between homozygous X178 and heterozygous alleles of *qLW4* were detected, revealing the completely dominant effect of *qLW4* (**Figures 4A,B** and Supplementary Figure S6). Moreover, the strong effect of *qLW4*, which was able to vary LW by 1.3 cm (in the RILs) to 3.8 cm (in the NILs), suggests that this QTL can serve as a valuable tool for the genetic modification of maize LW.

Pairwise comparisons of the LW of each mature leaf in these two NILs indicated that leaves of mature W964 plants were significantly wider than those of W968 (Supplementary Figure S8). LWs in maize V2 (two completely expanded leaves) seedlings exhibited significant differences as well (Supplementary Figure S9). Thus, *qLW4* may control the width of all leaves in maize plants, with this effect beginning with the emergence of first leaves. We examined the number of parallel veins in ear leaves of W964, W968, and W964/W968, but no difference was found among these lines (**Figure 4D**). This observation indicates that the effect of *qLW4* on LW involves variation of the interval width between adjacent parallel veins rather than a change in the number of parallel veins. Additional sub-cellular observation is needed to discern whether alteration of cell size or cell number, or both, is responsible for this change. The length of ear leaves among W964, W968, and W964/W968 individuals was also measured. No significant difference was found (**Figure 4C**), which indicates that *qLW4* does not influence maize leaf length.

### Prediction of Putative Candidate Genes for *qLW4*

Three putative candidate genes were identified in our fine-mapping interval, namely, GRMZM2G371159, GRMZM2G070723, and GRMZM2G070553, which, respectively, encode a Rapid ALkalinization Factor (RALF) family protein precursor, a peptide chain release factor protein, and a kelch repeat protein (**Table 2**). Expression analysis using public RNA-seq data^1^ revealed that GRMZM2G371159 showed no expressing signals in any leaf or shoot related tissues (Supplementary Figure S10). Further qPCR analysis of GRMZM2G070723 and GRMZM2G070553 indicated that both of them were differentially expressed between W964 and W968 in developing leaves (Supplementary Figure S11). Thus the real candidate gene for *qLW4* couldn’t be locked on yet. Nevertheless, 55 kb is still a large region for causal variation determination. The non-coding sequence variation, which always responsible for mild quantitative trait variation (Clark et al., 2006; Salvi et al., 2007), cannot be excluded from consideration. Thus, more work is needed for cloning this QTL.

**Table 2:** Candidate genes in fine-mapped *qLW4* region.

Chr	Start(bp)	End(bp)	Length	Gene ID	Annotation
4	3452127	3452588	461	GRMZM2G371159	Rapid ALkalinization Factor (RALF) family protein precursor
4	3453139	3459616	6477	GRMZM2G070723	Peptide chain release factor protein
4	3459901	3467312	7411	GRMZM2G070553	Kelch repeat protein

## Discussion

### Accelerated QTL Fine-Mapping by Re-sequencing of Large RILs in Maize

Bin mapping, which is based on bar-coded multiplexed re-sequencing, can increase linkage map resolution, thereby improving QTL mapping accuracy and saving time and resource (Huang et al., 2009). This method had been used in plants with simpler genomes than maize, such as rice and sorghum, to precisely identify several genes or QTLs with known functions (Huang et al., 2009; Zou et al., 2012). In the present study, we used a bin map with a 91-kb average physical interval between adjacent bins to precisely map *P1*, responsible for cob color variation, to a 700-kb interval, with the peak bin just overlapping with the gene body. For quantitative trait LW, we even directly mapped *qLW4* to a 270-kb interval, a range even much smaller than that of fine-mapped QTLs by several NILs (Chen et al., 2008; Tao et al., 2013b). In consideration of the density of our GBS-SNPs: 1 SNP average 102 kb, we inferred that deeper sequencing will be greatly helpful for improving our final mapping resolution. At the same time, we noted that the other three minor effect QTLs for LW still spanned a relatively large physical interval (2.87–8.35 Mb); in addition to using a higher-coverage genotype, we thus inferred that an enlarged population and additional environments for phenotyping would be beneficial for further mapping of these QTLs. Nevertheless, the interval size of less than 8.5 Mb corresponding to these minor effect QTLs is still smaller than that revealed by mapping studies using PCR-based markers or small populations (Reymond et al., 2004; Ku et al., 2010, 2012; Nikolic et al., 2011; Zheng and Liu, 2013; Guo et al., 2015).

Small populations may cause sampling bias—especially when individuals with extreme phenotypic values are present—thereby leading to misestimation of effects and erroneous localization of QTLs. The power of QTL detection is reported to increase with population size (Schön et al., 2004). The use of small populations may even result in misleading inferences (Vales et al., 2005). Thus, a larger population for QTL mapping is needed. Previously, we used 708 F_2_ individuals to conduct QTL mapping. Even though the number of recombinations in the F_2_ generation was limited, we still accurately mapped *r1* and *ba1* genes with high resolution because of the large population (Chen et al., 2014). Earlier, the RILs in this study were used in an investigation for fine-mapping of *Scmv1*, a major effect QTL responsible for resistance to maize sugarcane mosaic virus (Tao et al., 2013a). Historical accumulated recombination and saturated PCR-based markers allowed this locus to even be fine-mapped to a 59.21-kb interval. In the present study, using only 0.08×-coverage sequence data, we were able to narrow down *qLW4* to a 270-kb interval, further reflecting the power of large populations. Additionally, the use of a large population allowed rapid construction of NILs and a fine-mapping population, which is almost unattainable with small populations.

To date, many QTLs contributing to important agronomic trait variation have been cloned in maize, but their identification and fine-mapping has been a very convoluted process, such as *TAG1* (Doebley et al., 1990; Dorweiler et al., 1993; Wang et al., 2005) (the important domestication gene that influence glume induration), *VGT1* (Salvi et al., 2007) (an important locus involved in flowering time control), *qPH3.1* (Teng et al., 2013) and *QPH1* (Xing et al., 2015) (important plant height QTLs), *qHSR1* (Chen et al., 2008; Zuo et al., 2015) (a major effect QTL involved in resistance to head smut), etc.. Their initial localization always spanned a relatively large interval: 3–35 Mb. Their subsequent fine-mapping all experienced processes of more than three generations NILs construction, and genotyping and phenotyping of thousands of progenies. Several fine-mapping work even required a greater than five generations recombinant-derived progeny testing strategy (Yang et al., 2012), which necessitated the genotyping and phenotyping of thousands of individuals in each generation. These studies required researchers to expend enormous amounts of energy, resources, and time. In the present study, with help of a large population and a high-resolution bin map, we directly mapped *qLW4* to a ∼270-kb interval and quickly obtain NILs from only one generation’s work of screen and selfing. By using just a one-generation recombinant-derived progeny testing strategy, we were able to easily and rapidly pinpoint this QTL to a 55-kb interval, with vast savings in terms of time and cost.

Overall, our data empirically demonstrate the power of mapping QTLs by using a large population in combination with re-sequencing data, provide an efficient panel for high-resolution QTL mapping, and suggest a method for rapid fine-mapping or even cloning of major effect QTLs.

### *qLW4* May Represent a Novel LW-Controlling Locus in Maize

To date, several maize LW QTL mapping studies have been reported (Reymond et al., 2004; Ku et al., 2010, 2012; Nikolic et al., 2011; Zheng and Liu, 2013; Guo et al., 2015). None of these studies, however, uncovered a LW QTL around the 3.4-Mb position on chromosome 4; this was true even when a very powerful NAM population was used (Tian et al., 2011). We previously performed a genome-wide association study of the LW trait using a panel of 278 sequenced inbred lines (Jiao et al., 2012), but no significant signals were found around the *qLW4* region either (data not shown). In maize, it has been reported that rare variants could result phenotype variation, but almost unable to be mapped by association analysis (Xing et al., 2015). Thus we infer that *qLW4* might result from a rare variation, or a variation having low linkage disequilibrium with adjacent loci. HuangC, one parent of the RILs, is a distinct germplasm from United States (Jiao et al., 2012) and rare used in China, may partly explain the rare status of *qLW4*.

As mentioned in the introduction, identified genes directly affecting LW in maize mainly include WUSCHEL-like homeobox gene (*NS1* and *NS2*) (Nardmann et al., 2004) and Cellulose Synthase-Like D protein encoded gene (*CslD1*) (Hunter et al., 2012). Other reported genes possibly contributing to LW variation involved ta-siRNA related gene (*LBL1* and *RGD2*) (Nogueira et al., 2007; Douglas et al., 2010), protein kinase encoding gene (*LGN1* and *SLN1*) (Moon et al., 2013), AP2 transcription factor-like gene (*DIL1*) (Jiang et al., 2012), and D4-cyclin gene (*Asc1*) (Brooks et al., 2009). However, none of the three candidate genes in our fine-mapped QTL interval—GRMZM2G371159 (encoding a RALF family protein precursor), GRMZM2G070723 (encoding a peptide chain release factor protein), and GRMZM2G070553 (encoding a kelch repeat protein)—belong to any of the above gene families. We therefore suspect that *qLW4* is a novel LW-controlling locus in maize.

### Potential Application of *qLW4* in Maize Breeding

As reported, adaptation to a higher plant density has been more crucial to the improvement of maize yield over the past century than has been increasing grain yield per plant, thereby implying the importance of density breeding for modern maize (Hammer et al., 2009). Canopy structure influences light transmission and gas exchange in population-cultured maize, thus affecting its density tolerance (Cowling and Field, 2003; Stewart et al., 2003). While, LW undoubtedly affects canopy structure and is thus presumed to contribute to density tolerance, giving the importance of LW modification. The major effect LW QTL *qLW4* identified in this study was able to explain ∼25% of the phenotypic variation and varied LW for 1.3 (in RILs) to 3.8 (in NILs) cm, it is clearly an efficient tool for maize LW modification. This QTL has a completely dominant effect, has no additional influence on leaf length, represent its easy-to-use and specific feature for marker-assisted selection. In addition, the sole 55-kb fine-mapped interval and nearly saturated markers from this study can be used to exclusively introgress this locus into a target inbred line, with the genomic background otherwise relatively unchanged. Meanwhile, *qLW4* was isolated from a variety of Nongda108, one of the most important maize hybrids in China, further implying the feasibility of applying this QTL for maize breeding.

## Author Contributions

BW, WS, HZ, LE, and JL conceived and designed the experiments. BW, YZ, JZ, ZL, JG, WL, JC, CG, and XZ performed RILs DNA isolation and phenotype data collecting. HL constructed the sequence library of the RILs. BW and XD analyzed the sequencing data. BW and YZ conducted the QTL identification and further fine-mapping. BW, HZ, and WS wrote the paper.

## Conflict of Interest Statement

The authors declare that the research was conducted in the absence of any commercial or financial relationships that could be construed as a potential conflict of interest.

## Footnotes

1 http://qteller.com

## Abbreviations

GBS: genotyping-by-sequencing
Indel: insertion–deletion
LOD: logarithm (base 10) of odds
LW: Leaf width
NILs: near-isogenic lines
qPCR: Quantitative real-time PCR
QTL: Quantitative trait locus
RILs: recombinant inbred lines
SNPs: single-nucleotide polymorphisms
