# Supplementary material for: Identification and Fine-Mapping of a Major Maize Leaf Width QTL in a Re-sequenced Large Recombinant Inbred Lines Population
Source: Front Plant Sci. 2018 Feb 7;9:101. doi: 10.3389/fpls.2018.00101 (PMC5816676; doi:10.3389/fpls.2018.00101)
Supplement: Supplementary file 1 [file Data_Sheet_1.docx]

Supplementary Material

Identification and fine-mapping of a major maize leaf width QTL in a re-sequenced large recombinant inbred lines population

Baobao Wang^1§^, Yanbin Zhu^1§^, Jinjie Zhu^1§^, Zhipeng Liu^1§^, Han Liu^1^, Xiaomei Dong^1^, Jinjie Guo^1^, Wei Li^1^, Jing Chen^1^, Chi Gao^1^, Xinmei Zheng^1^, Lizhu E^1^, Jinsheng Lai^1^, Haiming Zhao^1*^& Weibin Song^1*^

^§^ These authors contributed equally to this work.

*** Correspondence:** Haiming Zhao: haiming223@163.com; Weibin Song: songwb@cau.edu.cn.

# Supplementary Figures and Tables

## Supplementary Figures

**
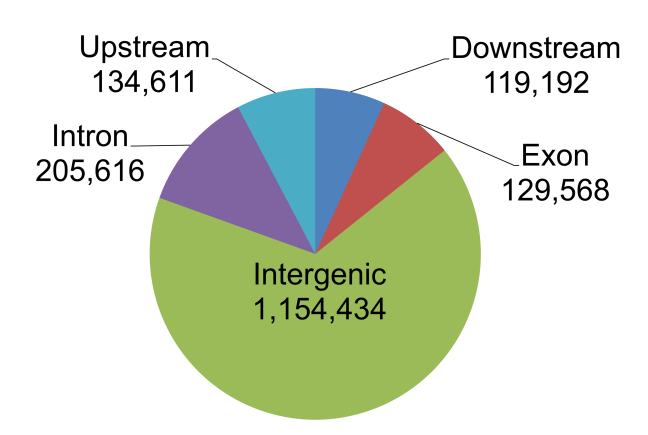
**

**Figure S1.** **Distribution of SNPs polymorphic between HuangC and X178 in different genomic regions.**

**
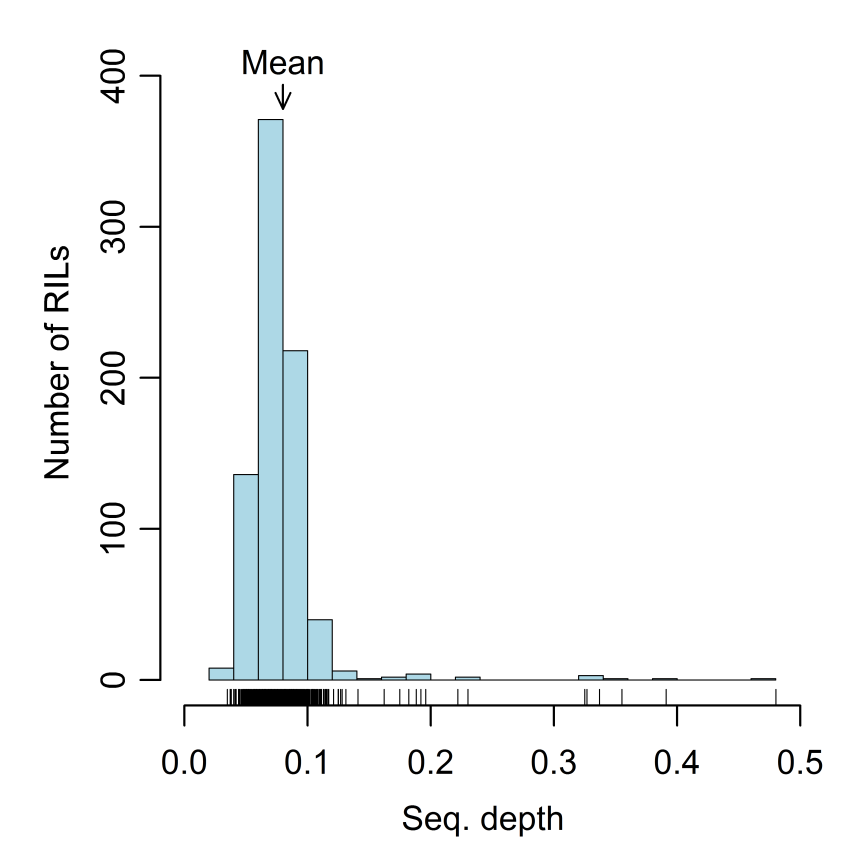
**

**Figure S2.** **Sequencing-depth profile of Nongda108 RILs.**

**
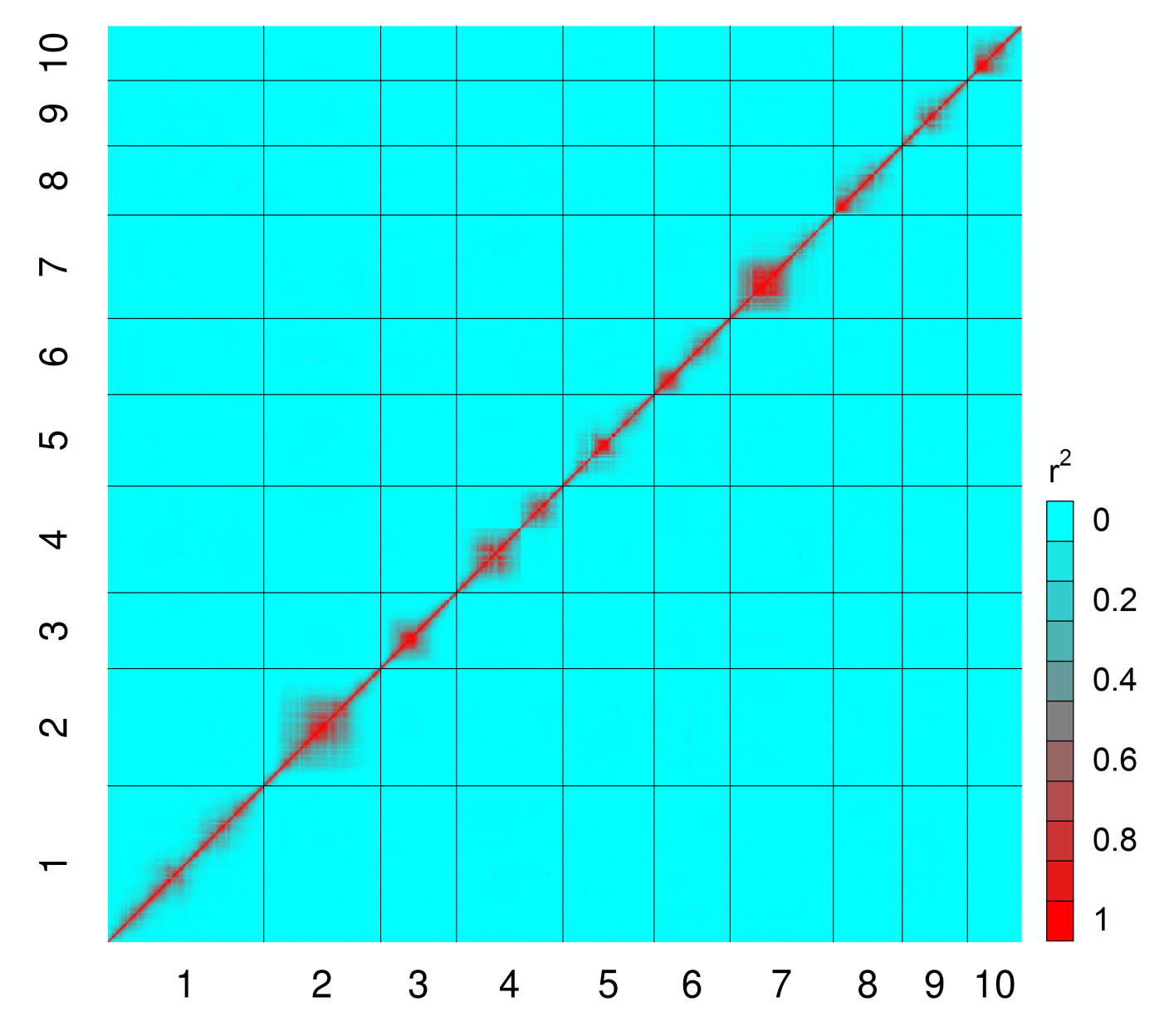
**

**Figure S3. Pair-wise linkage disequilibrium for all bin markers.** r^2^ of linkage disequilibrium were calculated by Plink software and displayed as intensity of colors in the legend.


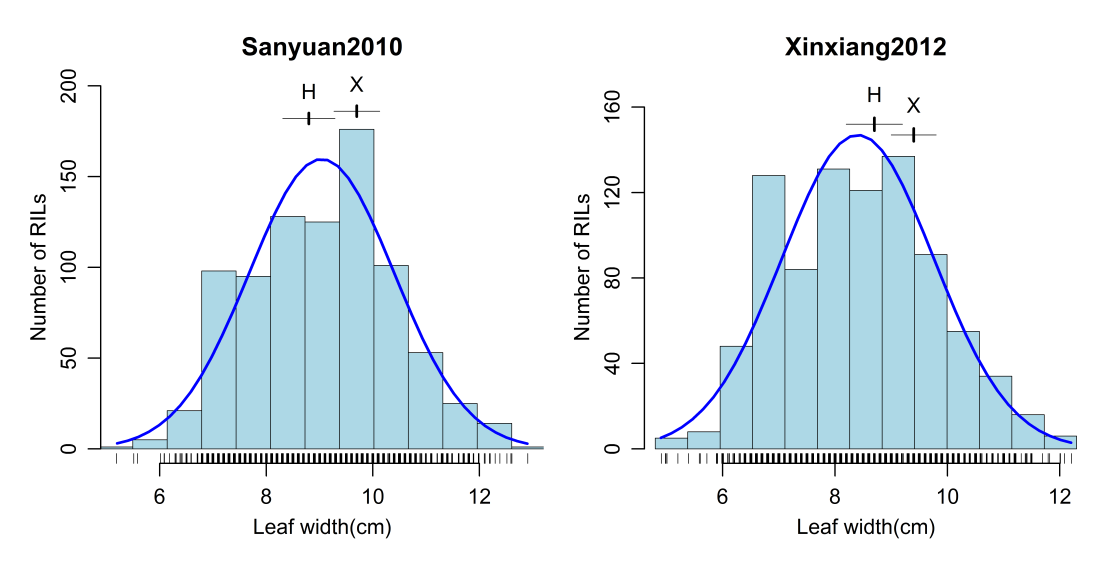


**Figure S4.** **Distribution of LWs in Nongda108 RILs in two locations.** H, mean of HuangC; X, mean of X178.

**
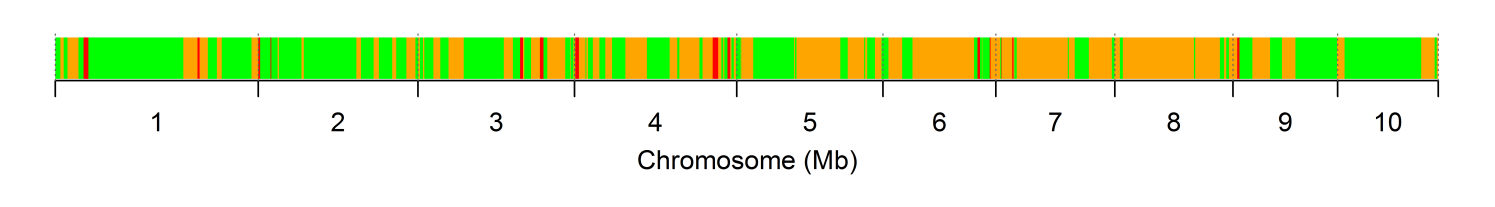
**

**Figure S5. Graphical representation of genotypes of R1504.** Green, X178 genotype; Orange, HuangC genotype; Red, heterozygous genotype.

**
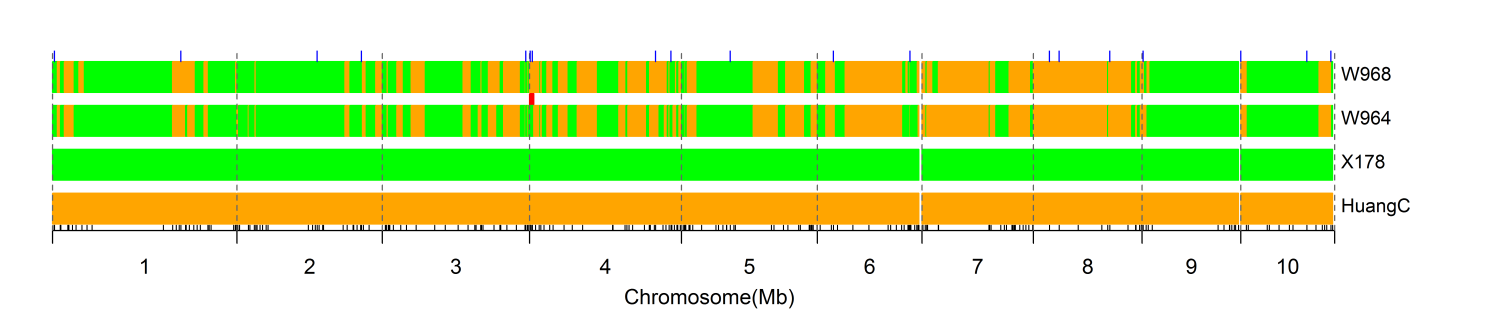
**

**Figure S6.** **Graphical representation of genotypes of HuangC, X178, W964 and W968.** GBS-SNPs and 17 PCR markers are shown below and above bars, respectively. The *qLW4* region is indicated by a red rectangle.

**
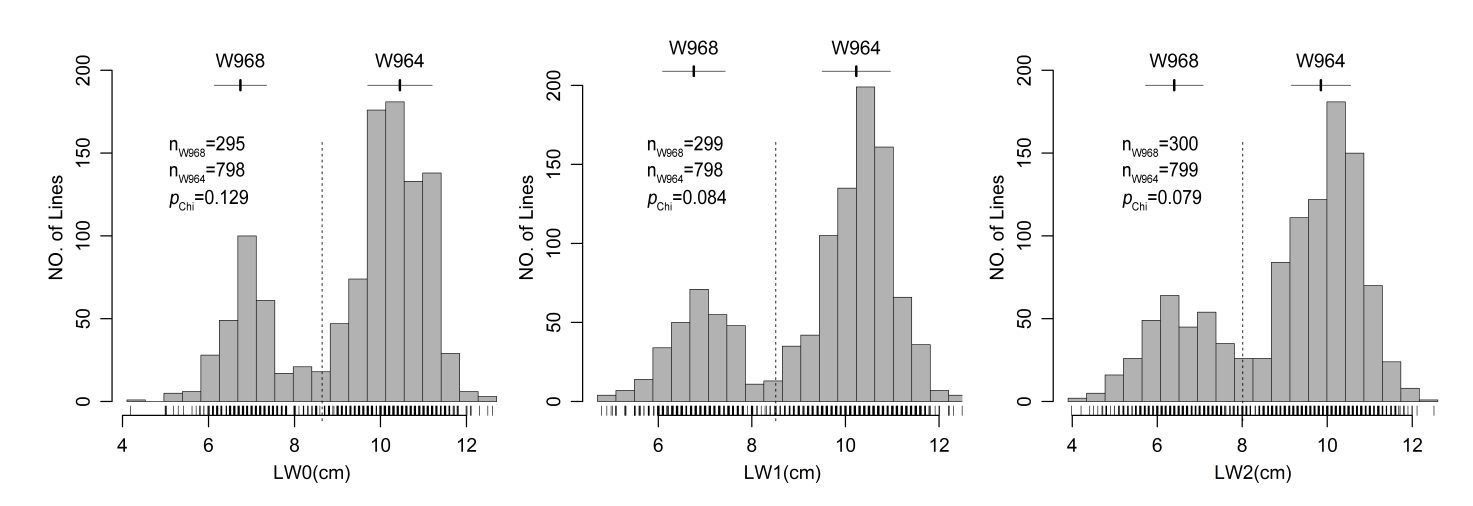
**

**Figure S7.** **Bimodal distribution of widths of ear leaves, 1^st^ upper leaves and 2^nd^ upper leaves in R1504 progenies.** Mean and S.D. of LWs of W964 or W968 are indicated by vertical and horizontal segments above histograms. LW was divided into two classes based on the W964-W968 midpoint. After counting the number of individuals in each class, a chi-square test was performed to determine whether the observed segregation ratio was in accordance with the expected 1:3 ratio. LW0, ear leaf width; LW1, 1^st^ upper leaf width; LW2, 2^nd^ upper leaf width.

**
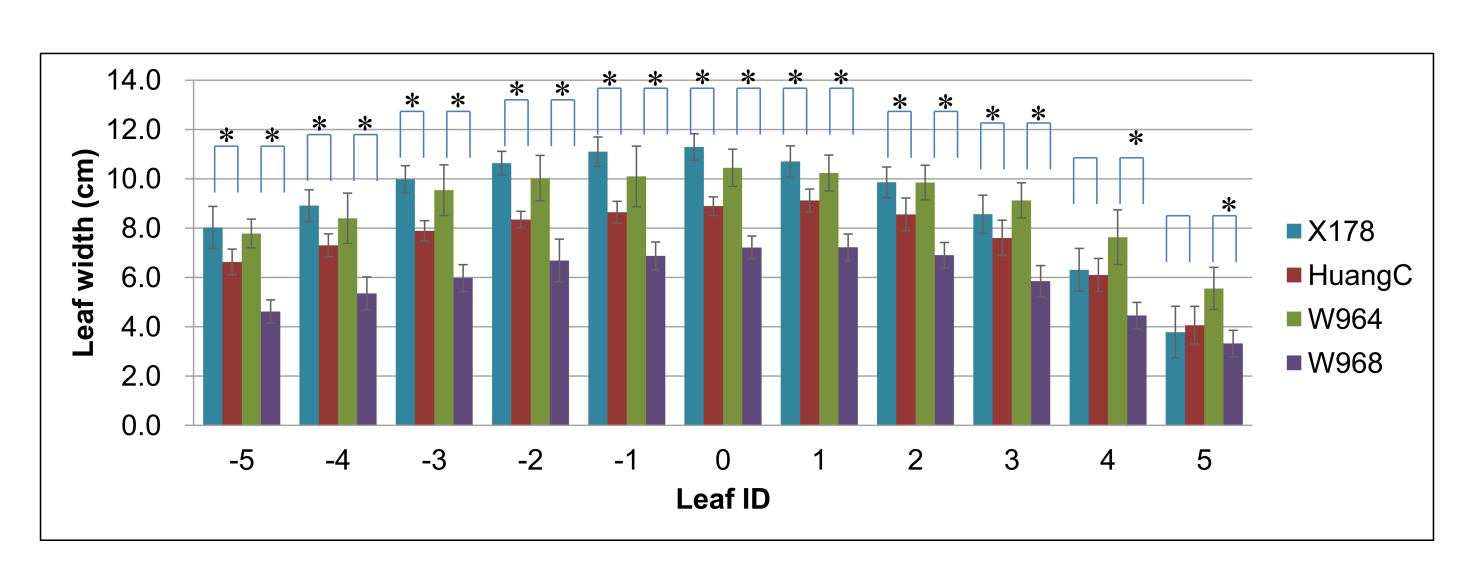
**

**Figure S8.** **Pairwise comparison of widths of corresponding leaves of mature plants of HuangC, X178, W968, and W964.** * indicates significant differences at the *p* < 0.05 level (*n* = 20). 0, ear leaf; 1 to 5, first to fifth upper leaves; -1 to -5, first to fifth lower leaves.

**
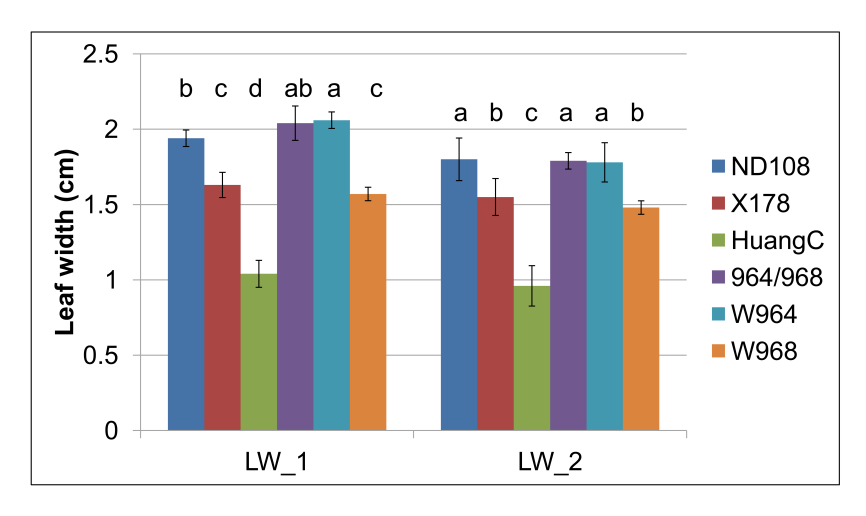
**

**Figure S9.** **Statistical analysis of widths of 1^st^ and 2^nd^ leaves of HuangC, X178, Nongda108, W968, W964, and W964/W968 maize seedlings at the V2 stage (only 2 expanded leaves)** (n=15).


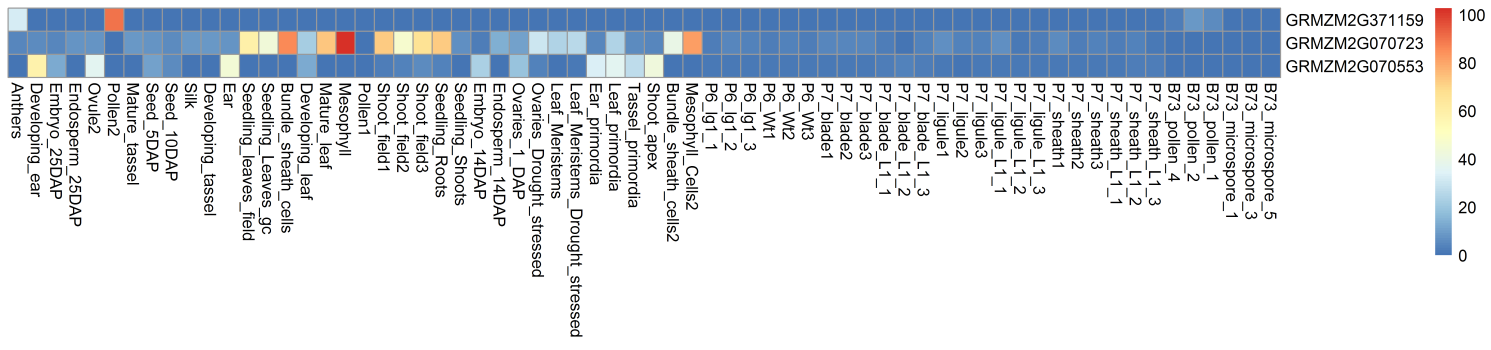
**Figure S10. Expression analysis of the three candidate genes by public RNA-seq data** (data from http://qteller.com).


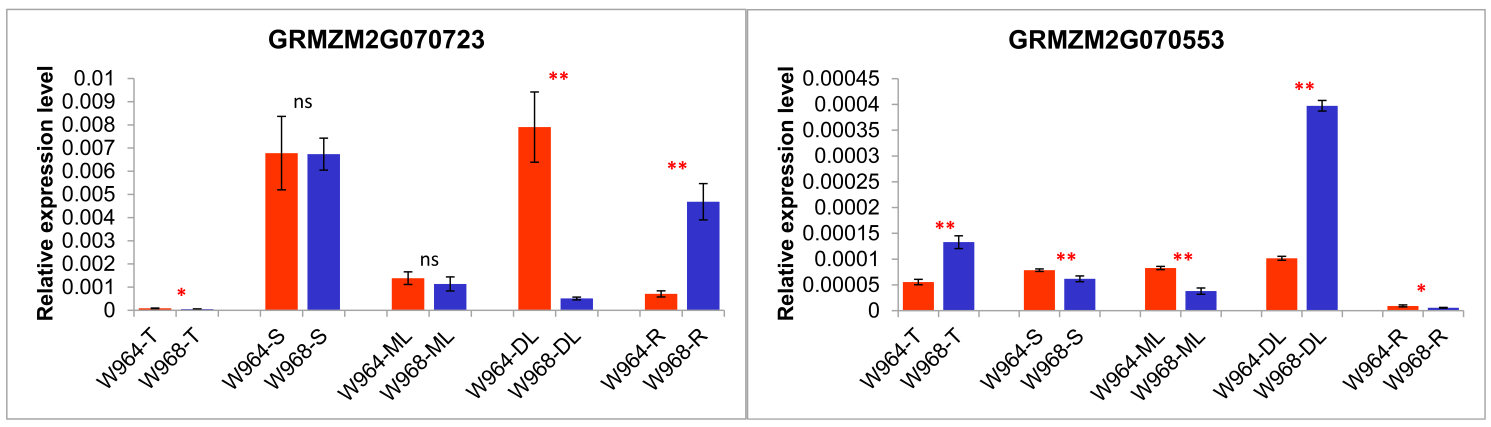


**Figure S11. Expression analysis of two candidate genes by qPCR method.** ns, no significant difference; *, 0.01<*p*<0.05; **, *p*<0.01. Suffix letters after W964 or W968: T, immature tassel; S, seedling shoot; ML, mature leaf; DL, developing leaf; R, seedling root.

## Supplementary Tables

**Table S1. Bin map summary.**

| Chr. | Bins |  |  |  | Linkage map (cM) | |  | Rec(cM/Mb)^e^ |
| --- | --- | --- | --- | --- | --- | --- | --- | --- |
|  | Number | Mean(Mb)^a^ | Max(Mb)^b^ |  | Total | Mean^c^ | Max^d^ |  |
| 1 | 3980 | 0.075 | 2.425 |  | 529.21 | 0.145 | 4.042 | 1.934 |
| 2 | 2686 | 0.088 | 2.28 |  | 366.624 | 0.13 | 5.956 | 1.485 |
| 3 | 1705 | 0.135 | 2.705 |  | 237.933 | 0.141 | 15.119 | 1.042 |
| 4 | 2966 | 0.081 | 1.135 |  | 402.916 | 0.142 | 12.039 | 1.755 |
| 5 | 2952 | 0.073 | 1.31 |  | 466.26 | 0.166 | 8.973 | 2.279 |
| 6 | 1681 | 0.099 | 1.305 |  | 202.739 | 0.121 | 2.346 | 1.22 |
| 7 | 2544 | 0.069 | 0.895 |  | 363.286 | 0.141 | 6.434 | 2.057 |
| 8 | 1698 | 0.103 | 1.4 |  | 249.458 | 0.128 | 8.851 | 1.244 |
| 9 | 2430 | 0.063 | 0.85 |  | 248.179 | 0.135 | 2.182 | 2.129 |
| 10 | 1201 | 0.123 | 1.13 |  | 163.822 | 0.136 | 2.264 | 1.108 |
| Total | 23843 | 0.091 | 2.705 |  | 3230.425 | 0.139 | 15.119 | 1.525 |

^a,b^ The average and maximal physical interval between the mid-point of adjacent bins, in Mb.

^c,d^ The average and maximal linkage interval of adjacent bins in cM.

^e^ Recombination ratio expressed as (length of linkage map)/(length of physical map), with a unit of cM/Mb.

**Table S2. Analysis of variance of LW across two locations.**

| Groups | Variance |
| --- | --- |
| Line | 1.430 |
| Line:Year | 0.090 |
| Line:Loc | 0.105 |
| Year | 0.132 |
| Loc | 0.069 |
| Residual | 0.298 |
|  |  |
| h^2^ | 0.893 |

**Table S3. Verification of *qLW4* by PCR markers.**

| Markers | Chromosome | Position(Mb) | P-value (t-test) | | |
| --- | --- | --- | --- | --- | --- |
|  |  |  | Sanyuan2010 | Xinxiang2012 | Sanya2013 |
| Indel_0.832 | 4 | 0.832 | 0.835 | 0.900 | 0.458 |
| Indel_1.459 | 4 | 1.459 | 0.833 | 0.845 | 0.379 |
| Caps_2.815 | 4 | 2.815 | 0.760 | 0.879 | 0.243 |
| Indel_2.836 | 4 | 2.836 | 0.845 | 0.979 | 0.275 |
| Caps_3.215 | 4 | 3.215 | 1.06E-03 | 2.30E-02 | 1.60E-03 |
| Caps_3.406 | 4 | 3.406 | 1.77E-07 | 3.36E-06 | 1.49E-06 |
| Caps_3.442 | 4 | 3.442 | 1.82E-07 | 1.79E-06 | 9.77E-07 |
| Caps_3.618 | 4 | 3.618 | 1.81E-03 | 1.89E-03 | 1.21E-03 |
| Indel_3.89 | 4 | 3.890 | 0.043 | 0.013 | 0.028 |
| Indel_4.1 | 4 | 4.100 | 0.513 | 0.946 | 0.630 |

**Table S4. Markers used in this study.**

| Name | Chr. | Position(Mb) | Forward primer | Reverse primer | Restriction enzymes |
| --- | --- | --- | --- | --- | --- |
| Indel_0.832 | 4 | 0.832070 | CCCAATGCACACGGTTACAT | CGCGAGGTTGCTTGTCATC |  |
| Indel_1.459 | 4 | 1.459304 | AAGGCTCCTTCTTCCTGGTG | ACAAGCTCCTTCCCTTTGGT |  |
| Caps_2.815 | 4 | 2.815805 | CCATCAACAACTGCCACCATC | TGAACCTGGGTCTTGTTTCTTTAC | HindIII |
| Indel_2.836 | 4 | 2.836808 | TGGTTCTTGGCTCCTCTGTCC | GGTGAGCGGCGTAGAGTTGA |  |
| Caps_3.215 | 4 | 3.215122 | ACTCGCCTAACCAAATCCCT | GTCAGTAAATATCAAGCCAACGTG | HphI |
| Caps_3.255 | 4 | 3.255505 | GTGCTCCATCTATTTGTTCTTATCCT | CCGTGGTGGACAACGAAGA | HapII |
| Indel_3.256 | 4 | 3.256625 | TCATGGCCAAGGGCAAGG | CGGTGATGTACGGGATGAAC |  |
| Caps_3.399 | 4 | 3.399430 | TCCCGTCAACCACCAAAGAG | CGACTTGACCATAGCCAACGA | AvaII |
| Caps_3.406 | 4 | 3.406856 | CTTCAGTCTTGGGACTTCTTCGT | CACGGAAGGCACTCGGTTTA | HapII |
| Caps_3.413 | 4 | 3.4136 | CACCCAACCCTGTCATCCTCT | TCGGTGACTGCGAGGACAA | AatII |
| Caps_3.442 | 4 | 3.442783 | TCACCGAGACGAGAAGACCG | AAGCCTCAGACCAGAAGCAAAT | AccI |
| Indel_3.451 | 4 | 3.451981 | ACAAGATGGCGGCAGGCA | GCTTCCTCCGATCCATCCG |  |
| Indel_3.453 | 4 | 3.453 | CAAATCACTGGGCATGGCA | TTGAGCCGACTGAGGAAGAGG |  |
| Indel_3.459492 | 4 | 3.459492 | GTGTGGCGTCTGGCGTGT | CGGAGCACGACTGTACGAGG |  |
| Indel_3.459603 | 4 | 3.459603 | AAAACAATATGATAGGAATCTGGGG | CGGGAGGCTGGAAAGGAC |  |
| Indel_3.459816 | 4 | 3.459816 | TTCCCCAGATTCCTATCATATT | GTATTTCGCAACTGGGACGTA |  |
| Indel_3.466475 | 4 | 3.466475 | TTGCGGCAGTAGGGTTCAG | CCGAACATCTTGGGCATTG |  |
| Indel_3.467037 | 4 | 3.467037 | CTCGATTTCACTTGACCGACG | AGTACACGGAGCAACGACGAG |  |
| Indel_3.467651 | 4 | 3.467651 | GATGCCTGGTCTGGTCTCAAGT | CATGGCAAAGGCGATTCACT |  |
| Indel_3.467953 | 4 | 3.467953 | AATCCACAACAGCTTTTAGTGCC | TTAGGAAAAGTCGCTATCTCCAATC |  |
| Indel_3.468695 | 4 | 3.468695 | AGCCCCATTTGGATCCTTG | TGCTTACATATGAATGATGCGGA |  |
| Indel_3.474215 | 4 | 3.474215 | TCGTGACGTCGCTCCTGAG | AGGCTGCCGCCCACGAAC |  |
| Caps_3.584979 | 4 | 3.584979 | GCTCACAAACACTAGAGGGACAA | AAGCAAGGGAAGTTTGGTAGAC | HaeIII |
| Caps_3.618 | 4 | 3.617925 | GGCTCCTCCGCTCCTCTAA | CCCTTCTCGGGTGGGATAG | DraI |
| Indel_3.74 | 4 | 3.740254 | TGACTGCCGTGCTCTGCTC | ACACATGGCATGAGGATGAGAG |  |
| Indel_3.89 | 4 | 3.89 | CACGAAAACAAAATTGTTGGA | TGGGAAGAATGTGGTCTGGAC |  |
| Indel_4.1 | 4 | 4.1 | AACTATTAGAGATAAAGGCTGA | AACTATGATTTGGAATGTGGAG |  |
| chr1-3.1 | 1 | 3.1 | ATGCTTAGAGGGTGTGTTAGGCTGA | TAATCAAATCGACGGACGGATTC |  |
| chr1-202.2 | 1 | 202.2 | CAGGAGACAGGAGGATGGCTACG | TCCTTTTTATAGATGCTCGGGGC |  |
| chr2-126.4 | 2 | 126.4 | GGGAGACTAATGACGAAGGAACG | TATAGTGGCACCCAAATAGAACCAA |  |
| chr2-196.2 | 2 | 196.2 | CTGGGCTCCGTCAGGCAGTG | CATCCTTCTCCGCTGCTTCCA |  |
| chr3-226.1 | 3 | 226.1 | CTCCCCAGCGTCACCACGTCTAA | AAACTCCTACGGATGCTTGCCTG |  |
| chr4-98.3 | 4 | 98.3 | AGGTCATCTCAGTCTCATCCGACAC | GTGACGGCAACGCAACAGTATCC |  |
| chr4-222.7 | 4 | 222.7 | CACAAACTGGACAAAGTTGATGC | TGACAACGCTTAAATGAACCTTGAT |  |
| chr5-76.6 | 5 | 76.6 | GGACGACGATCTACTGCGAGTTC | ATGGTAGTTTGGAATGGATGGAGTT |  |
| chr6-25.3 | 6 | 25.3 | GAGTTCAGTCAAGCAAGTCTTTTTTCG | AGCCAGAAGAAATTAAACGCATACG |  |
| chr6-145.9 | 6 | 145.9 | CTTCGGTAAGGCGACAGAATACG | GATATTTGGTTTGAAAAGGTGGAGTC |  |
| chr8-25.3 | 8 | 25.3 | CCCCACCATCGCAACGCTACT | GTTTTGTATGGCAGAGTTGGCTTTC |  |
| chr8-40.8 | 8 | 40.8 | AATCTGCACAACAGTGTCTTCATGG | TGATCCTGTGGACGGAGTAGCC |  |
| chr8-120.6 | 8 | 120.6 | CAGCCAGGACCTCGGCATAAAC | CGGTGAAGTTGAAGGTCTGGGAAG |  |
| chr9-1.465 | 9 | 1.465 | CCGTGGGGTAGTTGGAGCAATGT | CGACCACAGGACGGCACAGATAA |  |
| chr9-155.4 | 9 | 155.4 | GACGGTGATGCCCTTAGTGGT | CCGAAGCGAACGACCAGATAG |  |
| chr10-104.2 | 10 | 104.2 | TCTACTGCCCCCTGGCTCTG | TCTCTTGTGGCTACCGAGTTTTTGT |  |
| chr10-142.1 | 10 | 142.1 | GACTCCCAACGCAGAACCAAA | TCTCCCGCATTACCCACTACC |  |
| GRMZM2G070553-qPCR |  |  | GTGGGCATACGGTGATTAGAGC | GAGCATGAATATCATTCAGGGTCTT |  |
| GRMZM2G070723-qPCR |  |  | CCAGAGATACTGTGAGCGCAAC | AATATCTTTCGGATCAATAACCACA |  |
| EF1α-qPCR |  |  | TCTCAAGAACGGTGATGCTG | TGGGTCCTTCTTCTCCACAC |  |
